# Repeated oral administration of low doses of silver in mice: tissue distribution and effects on central nervous system

**DOI:** 10.1186/s12989-021-00418-x

**Published:** 2021-06-16

**Authors:** Camilla Recordati, Marcella De Maglie, Claudia Cella, Simona Argentiere, Saverio Paltrinieri, Silvia Bianchessi, Marco Losa, Fabio Fiordaliso, Alessandro Corbelli, Gianpaolo Milite, Federica Aureli, Marilena D’Amato, Andrea Raggi, Francesco Cubadda, Sabina Soldati, Cristina Lenardi, Eugenio Scanziani

**Affiliations:** 1 Dipartimento di Medicina Veterinaria (DIMEVET), Università degli Studi di Milano, 26900 Lodi, Italy; 2 Fondazione Unimi, 20139 Milan, Italy; 3 Dipartimento di Fisica, Università degli Studi di Milano, 20133 Milan, Italy; 4 Unit of Bio-imaging, Department of Molecular Biochemistry and Pharmacology, Istituto di Ricerche Farmacologiche Mario Negri IRCCS, 20156 Milan, Italy; 5 Scientific Consultant, Udine, Italy; 6 Istituto Superiore di Sanità - National Institute of Health, 00161 Rome, Italy; 7 Centro Interdisciplinare Materiali e Interfacce Nanostrutturati (CIMAINA), Università degli Studi di Milano, 20133 Milan, Italy

**Keywords:** Silver nanoparticles, Silver acetate, Oral administration, Tissue distribution, Toxicity, Central nervous system, Blood brain barrier, Mouse

## Abstract

**Background:**

Widespread use of silver in its different forms raises concerns about potential adverse effects after ingestion, the main exposure route for humans. The aim of this study was to investigate in CD-1 (ICR) male mice the tissue distribution and in vivo effects of 4-week oral exposure to 0.25 and 1 mg Ag/kg bw 10 nm citrate coated silver nanoparticles (AgNPs) and 1 mg Ag/kg bw silver acetate (AgAc) at the end of treatment (EoT) and after 4 weeks of recovery.

**Results:**

There were no treatment-related clinical signs and mortality, and no significant effects on body and organ weights at the EoT and after recovery. Treatment-related changes in hematology and clinical chemistry were found after recovery, the most relevant being a dose-dependent lymphopenia and increased triglycerides in AgNP-treated mice, and increased levels of urea in all treated groups, associated with decreased albumin only in AgAc-treated mice. At the EoT the highest silver concentration determined by Triple Quadrupole ICP-MS analysis was found in the brain, followed by testis, liver, and spleen; much lower concentrations were present in the small intestine and kidney. Tissue silver concentrations were slightly higher after exposure to AgAc than AgNPs and dose dependent for AgNPs. After recovery silver was still present in the brain and testis, highlighting slow elimination. No histopathological changes and absence of silver staining by autometallography were observed in the organs of treated mice. At the EoT GFAP (astrocytes) immunoreactivity was significantly increased in the hippocampus of AgNP-treated mice in a dose-dependent manner and Iba1 (microglial cells) immunoreactivity was significantly increased in the cortex of 1 mg/kg bw AgNP-treated mice. After recovery, a significant reduction of Iba1 was observed in the cortex of all treated groups. TEM analysis of the hippocampus revealed splitting of basement membrane of the capillaries and swelling of astrocytic perivascular end-feet in 1 mg/kg bw AgNP- and AgAc-treated mice at the EoT.

**Conclusions:**

Our study revealed accumulation and slow clearance of silver in the brain after oral administration of 10 nm AgNPs and AgAc at low doses in mice, associated with effects on glial cells and ultrastructural alterations of the Blood-Brain Barrier.

**Supplementary Information:**

The online version contains supplementary material available at 10.1186/s12989-021-00418-x.

## Background

Silver in its different forms, including silver ions and nanoparticles, is widely used because of its antibacterial activity. The antimicrobial properties of silver compounds have been studied for decades, and many studies suggest that silver disrupts microbial cell wall permeability and affects cellular respiration [1, 2], even though its multifaceted mechanism of antibacterial action is not yet fully elucidated [3, 4].

Recently, the application of silver in nanoform has increased and silver nanoparticles (AgNPs) are one of the leading nanomaterials on the market [5–7]. As a consequence of the nanosize the physico-chemical properties of AgNPs are different from those of the conventional counterparts (soluble Ag), affecting their fate and enhancing their biological activity [7, 8]. The specific characteristics of AgNPs have led to their application in many consumer products (food production and packaging, cosmetics, textiles) and in medicine (surgery, implants and medical devices, wound therapy) [7, 9, 10]. Recently, application of AgNPs has expanded to emerging fields such as drug delivery and diagnosis (e.g., antiviral and anticancer agents, photosensitizers) [11–13].

The widespread use of AgNPs leads to increased human exposure through ingestion, skin contact, and inhalation and raises concern about potential health effects [7, 14, 15]. Considering the different applications of AgNPs, the oral route of exposure emerges as the most significant, not only for the growing number of food-related uses but also as a consequence of unintentional ingestion via handling of consumer products incorporating AgNP, especially in children [7]. In order to characterize the risks of oral exposure to AgNPs for the human health, the assessment of AgNPs toxicity via in vitro and in vivo models is essential [16].

Ingested AgNPs pass through different physiological and physicochemical environments, which may alter their properties before they reach the intestinal cells and are eventually absorbed. Translocation of particles through the intestinal wall is a multistep process, involving diffusion through the mucus lining of the gut wall and absorption [17]. In vitro digestion models elucidated the changes AgNPs undergo in the gastrointestinal tract and concluded that AgNPs after ingestion can reach the intestinal epithelial cells with marginal aggregation and largely maintaining their initial size and composition [18, 19]. In addition, studies performed on intestinal epithelium models revealed that AgNPs can be internalized in enterocytes and pass the intestinal barrier [20, 21]. The same in vitro models provided insight into AgNPs toxicity in intestinal cells revealing that AgNPs can alter the proliferation rate and induce inflammation, increased generation of reactive oxygen species (ROS), and possibly DNA damage (reviewed by [22, 23]).

A few in vivo studies in rodents investigated the fate and effects of AgNPs after oral exposure, even though an overall interpretation is complicated by differences in study design and in the physicochemical properties of the test materials. Repeated oral administration of variably sized AgNPs and Ag ions at different doses resulted in the distribution of Ag in a wide range of organs [24–29]. Ag was identified microscopically along the small intestine surface, in the lamina propria, and in the submucosa [24, 26]. Using transmission electron microscopy (TEM) Ag granules (containing also selenium and sulfur) were identified in the epithelial basal lamina, in the macrophages of the lamina propria, and in the submucosa of the ileum of rats exposed to both AgNP and silver acetate (AgAc) [26].

Adverse effects reported in AgNP oral dosing studies were overall mild and evident at doses of 125 mg/kg bw and above [16, 30]. The observed effects included weight loss, increase in both serum pro-inflammatory and anti-inflammatory cytokines, damage to the enterocyte microvilli as well as intestinal glands, increased cholesterol, hepatic effects, such as increase in alanine aminotransferase (ALT) and aspartate aminotransferase (AST), and poorly defined histological changes [24, 25, 28–32].

Recent studies revealed effects of AgNPs on the central nervous system (CNS), after single or repeated administration through different routes. Blood brain barrier (BBB) functional alteration leading to brain edema was observed in mice and rats after single intravenous (30 mg/kg bw) or intraperitoneal (50 mg/kg bw) administration of 50–60 nm AgNPs [33, 34]. Single subcutaneous injection of AgNPs (50–100 nm) in rats at a dose of 62.8 mg/kg bw induced BBB destruction, astrocyte swelling, and neuronal degeneration, as revealed by TEM analysis [35]. Similar ultrastructural alterations were observed also after single intravenous administration of 5 mg/kg bw of 7 nm AgNPs in rats that resulted in decreased gene expression of claudin 4 (a tight junction protein) and ultrastructural detection of astrocyte foot swelling, neuron shrinkage and AgNP-like particles [36]. A dose-dependent induction of apoptosis, further supported by the increased Bax/Bcl-2 ratios, was observed in the hippocampus after the intraperitoneal administration of 14 nm AgNPs at 100, 200, and 400 mg/kg bw for 5 days in rats [37].

Repeated oral administration of 14 nm AgNPs in rats at a dose of 2.25, 4.5 and 9 mg/kg bw for 14 and 28 days affected neurotransmitters (5-HT and dopamine) concentration; effects depended on the dose and length of exposure [38]. Repeated oral administration of 10 nm AgNPs in rats at a dose of 0.2 mg/kg bw for 14 days induced considerable synaptic damage (mainly in the hippocampus) and resulted in morphological abnormalities in myelin sheaths and altered expression of myelin proteins [39, 40]. A similar protocol (AgNP size, dose and treatment duration) induced oxidative stress in rat brain [41, 42] and autophagy associated with mitochondrial ultrastructural changes (swelling and cristolysis) [43]. Repeated oral administration of 3–10 nm AgNPs at a dose of 1 and 10 mg/kg for 14 days in rats caused ultrastructural changes (neuron shrinkage, astrocyte cytoplasmic and foot swelling, extravascular lymphocytes accumulation), slightly increased gene expression of cadherin 1 and claudin-1, and increased levels of IL-4, suggesting a role of inflammation in central nervous system damage [44].

Considering the widespread application of AgNPs leading to increased human exposure and the need to characterize potential hazards, the aim of this study was to evaluate the effects of repeated (4 weeks) oral administration of low doses of 10 nm AgNPs in mice and compare them with those induced by Ag ions. The size of the nanoparticles investigated in this study was selected following findings of markedly increased toxicity compared to larger AgNPs after single intravenous administration in mice [45], and because oral exposure to 10 nm particles is a relevant scenario for humans both via consumption of E174-containing foods and ingestion of particles released from food contact materials, also considering that AgNPs are prone to dissolution with associated particle size reduction [46].

The design of the present study included the characterization of AgNP suspensions before and after use, assessment of tissue distribution by determining silver concentrations in main organs, hematology and serum chemistry, and histopathological, histochemical, immunohistochemical and ultrastructural examinations to evaluate the occurrence of adverse effects, with focus on the CNS.

## Results

### Physicochemical characterization of silver nanoparticles

AgNPs are known to degrade over time as a function of a number of parameters, including particle size, concentration and exposure to oxygen [47, 48]. In order to ensure that observed effects are due to AgNPs and not to their degradation products, characterization of stock AgNPs and their dispersions over the time course of the experiments represents a fundamental step in nanotoxicological studies.

**Fig. 1:**
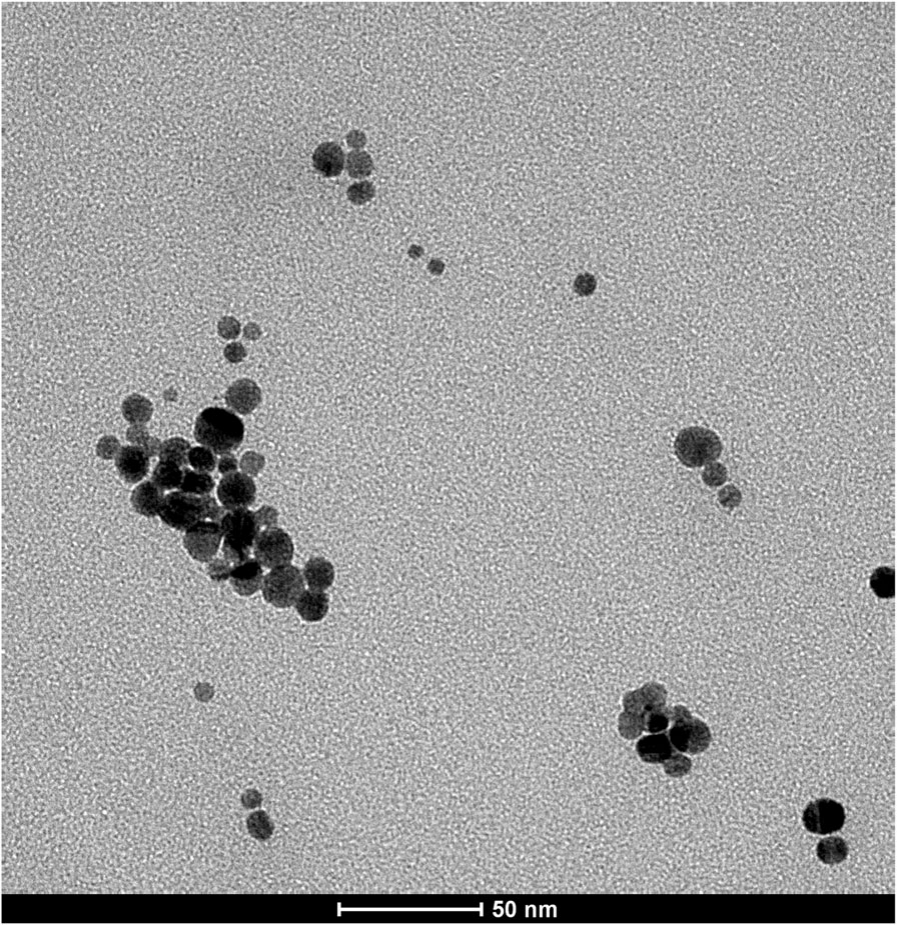
Representative TEM image of 10 nm CT-coated AgNPs. Particles were spherical in shape and mainly present as primary particles or loose agglomerates; no stable aggregates were visible (scale bar = 50 nm)

The TEM analysis confirmed that the AgNPs were spherical in shape and with a size of 10.0 ± 2.6 nm (Fig. 1), in accordance with that reported by the manufacturer (9.4 ± 1.7 nm).

**Fig. 2:**
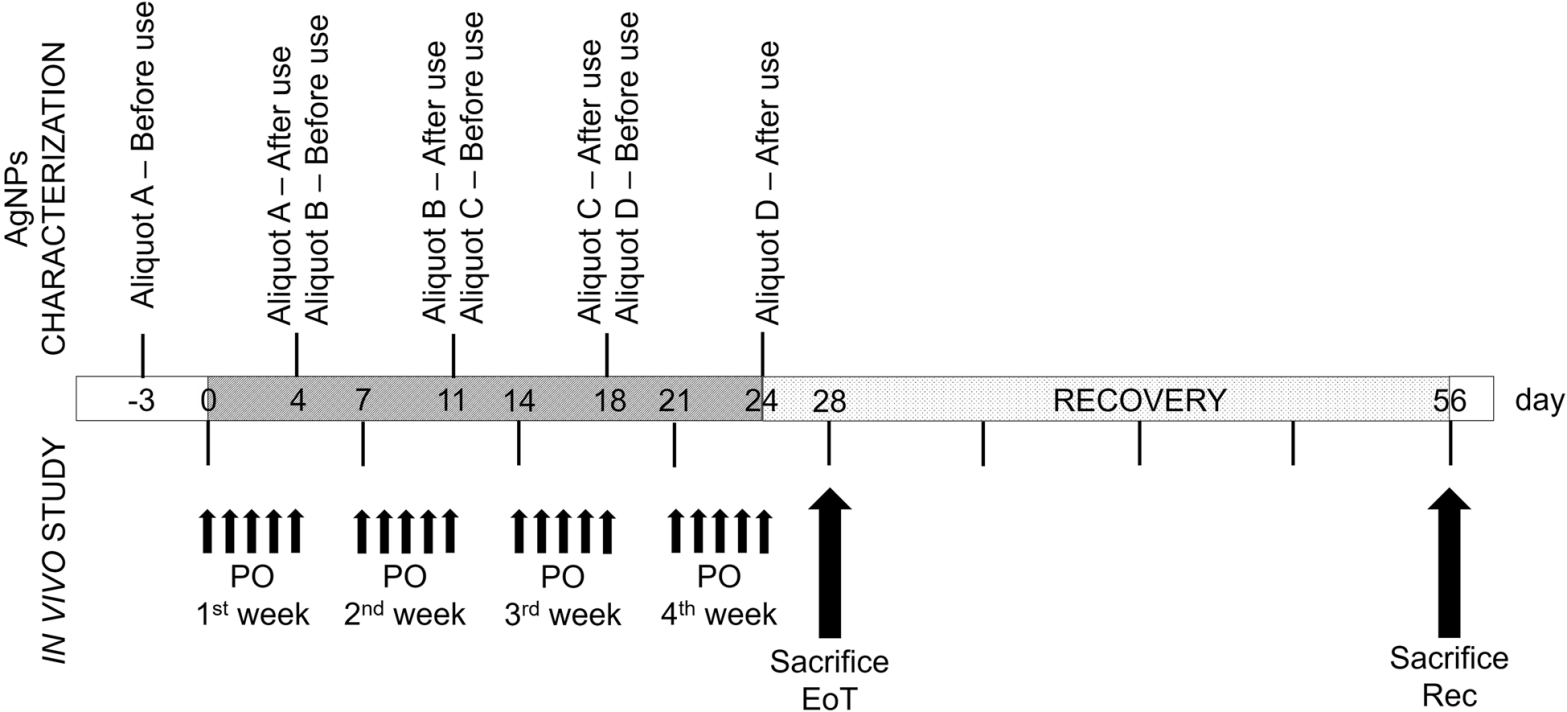
Experimental design. PO = per os administration; EoT = end of treatment; Rec = recovery

Aliquots of the dispersions for AgNP administration were characterized by DLS and/or UV-vis spectroscopy before the beginning of each dosing week (day − 3, 4, 11, 18), and at the end of each dosing week (day 4, 11, 18, 25) on the residual volumes (Fig. 2). Results are reported in Table 1.

**Table 1:** Physicochemical characterization of silver nanoparticles

AgNP aliquot^**b**^	Before use^a^					After use^a^	
	DLS^**c**^			UV-Vis^d^		UV-Vis^**d**^	
	Mean size (nm)	Peak (nm)	PdI	λ_**max**_(nm)	H_**max**_ (A.U.)	λ_**max**_(nm)	H_**max**_ (A.U.)
A	14.17 ± 0.23	15.30 ± 0.46	0.103 ± 0.047	391.89 ± 0.33	163.69 ± 0.60	391.44 ± 0.53	65.58 ± 12.39
B	14.46 ± 0.42	15.42 ± 0.66	0.129 ± 0.063	391.89 ± 0.33	161.35 ± 1.68	391.22 ± 0.44	130.52 ± 7.18
C	19.42 ± 7.42	15.21 ± 1.27	0.242 ± 0.063	391.00 ± 0.00	173.51 ± 4.62	391.00 ± 0.00	156.47 ± 2.51
D	14.59 ± 0.14	15.44 ± 0.96	0.159 ± 0.67	391.00 ± 0.00	161.31 ± 4.18	392.00 ± 0.00	158.61 ± 3.86

^a^ Quality control of AgNPs suspensions performed before the beginning of each dosing week (day −3, 4, 11, 18, “before use”), and at the end of each dosing week (day 4, 11, 18, 24, “after use”)

^b^Aliquots A, B, C, D were used in the 1st, 2nd, 3rd and 4th dosing week of the study, respectively

^c^For DLS analyses, the mean size of AgNPs is expressed in terms of hydrodynamic diameter and the maximum intensity peak is reported to describe samples having multimodal distributions, with the polydispersity index (pdI) providing a measure of particles uniformity

^d^For UV-Vis analyses, the maximum wavelength (λ_max_, i.e. the wavelength corresponding to the highest absorbance of AgNPs) as well as the maximum absorbance value (H_max_) are reported and are expressed as nanometer (nm) and arbitrary units (A.U.), respectively. The optical properties reported by the manufacturer NanoComposix (San Diego, USA) were: λmax 389 nm, H_max_ 167.37

Considering the AgNP aliquots tested at days − 3, 4, 11 and 18, the hydrodynamic diameter was comparable, within the experimental error, to that reported by the manufacturer (14.5 nm). The larger mean size and associated error (19.42 ± 7.42 nm) together with a higher polydispersity (0.242) of the nanoparticles tested at day 11 suggested the presence of few agglomerates. However, all the aliquots showed the λ_max_ of the UV-Vis absorption peak at around 391 nm, systematically red-shifted of about 2 nm and slightly less intense with respect to the data of manufacturer (λ_max_ 389 nm, H_max_ 167.37), results ascribable to different instrument calibrations. These analyses proved that AgNPs were substantially not aggregated after producer delivery and therefore suitable for the in vivo experiments.

At the end of each dosing week, residual aliquots were analysed to assess their agglomeration state. Characterization was performed only by UV-vis spectroscopy due to the small volume of residual AgNPs aliquots. As shown in Table 1, formation of secondary particles was appreciable after the first dosing week (aliquot A, analysed at day 4), suggesting that the use of a single dispersion for five consecutive administrations was not optimal. Accordingly, the remaining AgNP aliquots (aliquots B, C, and D) for the following dosing weeks were further split into three parts. This strategy was successful, since the optical properties of residual aliquots after these dosing periods were in agreement with those reported by the manufacturer.

Total silver measured in the AgNP suspension was found to be 0.80 mg/ml instead of 1.01 mg/ml, i.e. the value declared by the manufacturer. Accordingly, the actual delivered doses were 20% lower than the nominal ones.

### Animal behavior, body and organ weights

During the treatment and the recovery period, all mice appeared healthy, and no abnormal behavior was observed in mice treated with AgNPs at the nominal doses of 0.25 and 1.0 mg/kg bw and AgAc at the dose of 1.55 mg/kg bw (containing the equivalent dose of 1 mg Ag/kg bw). There was no significant difference in the body weights, body weight gain, and absolute organ weights between treated and control mice at the EoT and at the end of the recovery period (Table 2).

**Table 2:** Body weight, body weight gain, and organ weight at the end of treatment (EoT) and recovery (Rec) period (*n* = 6). Data are expressed as mean value ± SD

Time point	Group	Body weight at sacrifice (g)	Body weight gain at sacrifice (%)	Liver (g)	Spleen (g)	Kidneys (g)
EoT	Control (vehicle)	35.58 ± 2.73	21.19 ± 6.19	2.50 ± 0.30	0.12 ± 0.05	0.66 ± 0.07
	AgNP 0.25 mg/kg bw^a^	37.41 ± 2.28	30.76 ± 9.74	2.45 ± 0.30	0.12 ± 0.06	0.70 ± 0.07
	AgNP 1 mg/kg bw^a^	35.93 ± 3.22	28.96 ± 6.,86	2.50 ± 0.29	0.10 ± 0.01	0.69 ± 0.07
	AgAc 1.55 mg/kg bw	36.15 ± 1.60	21.24 ± 9.94	2.42 ± 0.31	0.12 ± 0.,03	0.66 ± 0.08
Rec	Control (vehicle)	42.60 ± 3.33	47.2 ± 5.80	2.15 ± 0.30	0.10 ± 0.01	0.71 ± 0.05
	AgNP 0.25 mg/kg bw^a^	42.62 ± 2.04	52.87 ± 9.89	2.24 ± 0.21	0.12 ± 0.02	0.71 ± 0.07
	AgNP 1 mg/kg bw^a^	41.27 ± 2.08	47.30 ± 7.29	2.48 ± 0.20	0.11 ± 0.01	0.73 ± 0.05
	AgAc 1.55 mg/kg bw^b^	40.12 ± 4.23	43.83 ± 14.12	2.25 ± 0.33	0.13 ± 0.05	0.69 ± 0.06

^a^Nominal dose (actual delivered doses were 0.20 and 0.80 mg/kg bw)

^b^Containing the equivalent dose of 1 mg Ag/kg bw

### Hematology and clinical chemistry

Hematological and clinical chemistry results are summarized in Tables 3 and 4. At the EoT, treatment with AgNPs did not induce haematological changes. Clinical chemistry revealed a decrease in the concentration or activity of GLDH, Urea, Creatinine and Triglycerides in mice treated with AgNP at the nominal dosage of 0.25 mg/kg bw. Treatment with AgAc did not induce haematological or biochemical changes at the EoT, except for a slight increase in the MCH.

**Table 3:** Hematological values at the end of treatment (EoT) and the recovery (Rec) period (*n* = 6). Data are expressed as mean value ± SD

Time point	Group	WBC x	RBC x	Hb	Ht	MCV	MCH	MCHC	Plt x	Neutr x	Lymph x	Mono x	Eos x
		10^3^/μL	10^6^/μL	g/dL	%	fL	pg	%	10^3^/μL	10^3^/μL	10^3^/μL	10^3^/μL	10^3^/μL
**EoT**	Control (vehicle)	8.78 ± 1.73	8.13 ± 0.78	11.27 ± 1.14	44.85 ± 2.97	55.27 ± 2.24	13.85 ± 0.29	25.05 ± 1.04	1586.50 ± 318.02	1.15 ± 0.61	7.34 ± 1.41	0.27 ± 0.14	0.03 ± 0.05
	AgNP 0.25 mg/kg bw^1^	8.32 ± 1.59	8.40 ± 0.37	11.52 ± 0.46	46.23 ± 2.42	54.92 ± 0.80	13.68 ± 0.08	24.93 ± 0.34	1448.17 ± 605.28	1.19 ± 0.38	6.40 ± 0.88	0.58 ± 0.54	0.15 ± 0.24
	AgNP 1.00 mg/kg bw^1^	10.05 ± 2.93	8.77 ± 0.32	12.22 ± 0.52	48.93 ± 2.06	55.65 ± 1.33	13.88 ± 0.42	24.98 ± 0.36	1480.00 ± 305.90	0.89 ± 0.35	8.76 ± 2.97	0.33 ± 0.23	0.08 ± 0.11
	AgAc 1.55 mg/kg bw^2^	9.33 ± 2.59	8.23 ± 0.65	11.65 ± 0.87	46.63 ± 3.31	56.63 ± 1.35	14.17 ± 0.24^a^	25.00 ± 0.20	1409.50 ± 360.08	1.90 ± 2.53	6.76 ± 3.11	0.54 ± 0.47	0.15 ± 0.13
**Rec**	Control (vehicle)	10.48 ± 1.99	8.55 ± 0.48	12.07 ± 0.88	48.60 ± 3.24	56.87 ± 1.18	14.12 ± 0.35	24.82 ± 0.66	1390.67 ± 392.04	1.15 ± 0.53	8.67 ± 1.36	0.58 ± 0.31	0.09 ± 0.11
	AgNP 0.25 mg/kg bw^1^	7.98 ± 1.56 ^a^	8.83 ± 0.60	12.47 ± 0.73	50.93 ± 2.58	57.70 ± 1.74	14.10 ± 0.50	24.47 ± 0.39	999.33 ± 209.96	1.04 ± 0.24	6.48 ± 1.48 ^a^	0.44 ± 0.21	0.05 ± 0.06
	AgNP 1.00 mg/kg bw^1^	6.80 ± 2.55*^/ a^	8.12 ± 0.53	11.58 ± 0.56	47.23 ± 2.29	58.25 ± 2.71	14.28 ± 0.48	24.53 ± 0.87	1069.17 ± 379.94	1.16 ± 0.33	5.17 ± 2.26*^/a^	0.42 ± 0.31	0.06 ± 0.09
–	AgAc 1.55 mg/kg bw^2^	8.50 ± 4.82	9.08 ± 0.39	12.62 ± 0.37	50.77 ± 1.33	56.03 ± 1.25	13.93 ± 0.41	24.85 ± 0.29	933.33 ± 155.88	2.05 ± 1.75	5.82 ± 2.85 ^a^	0.57 ± 0.52	0.06 ± 0.09

^1^ Nominal dose (actual delivered doses were 0.20 and 0.80 mg/kg bw)

^2^Containing the equivalent dose of 1 mg Ag/kg bwStatistical significance: * = *p* < 0.05; vs Control (Kruskal-Wallis test, followed by Dunn’s multiple comparison test); a = *p* < 0.05 vs Control (Mann-Whitney U test)

**Table 4:** Serum chemistry values at the end of treatment (EoT) and the recovery (Rec) period (*n* = 6). Data are expressed as mean value ± SD

Time point	Group	GLDH	Urea	Creatinine	Triglycerides	ALT	Albumin	Total protein
		(U/L)	(mg/dL)	(mg/dL)	(mg/dL)	(U/L)	(mg/dL)	(mg/dL)
**EoT**	Control (vehicle)	8.00 ± 3.51	39.33 ± 3.14	0.32 ± 0.03	154.83 ± 48.40	37.17 ± 28.25	2.31 ± 0.24	5.27 ± 0.79
	AgNP 0.25 mg/kg bw^1^	4.52 ± 2.41^a^	33.83 ± 4.92 ^a^	0.28 ± 0.01 ^a^	93.50 ± 35.49 ^a^	18.17 ± 6.31	2.40 ± 0.22	4.60 ± 0.29
	AgNP 1.00 mg/kg bw^1^	11.28 ± 3.75	43.83 ± 5.00	0.35 ± 0.11	157.33 ± 36.19	17.83 ± 5.38	2.45 ± 0.29	5.34 ± 0.42
	AgAc 1.55 mg/kg bw^2^	13.32 ± 11.51	34.83 ± 4.02	0.30 ± 0.04	126.50 ± 100.74	24.33 ± 22.72	2.30 ± 0.16	4.54 ± 0.47
**Rec**	Control (vehicle)	25.25 ± 12.92	35.83 ± 3.76	0.34 ± 0.02	171.67 ± 105.08	91.83 ± 88.37	2.12 ± 0.14	5.08 ± 0.91
	AgNP 0.25 mg/kg bw^1^	21.13 ± 6.99	45.00 ± 4.56^b^	0.37 ± 0.04	233.00 ± 49.78	64.33 ± 52.47	2.43 ± 0.18 ^b^	5.76 ± 0.83
	AgNP 1.00 mg/kg bw^1^	38.62 ± 22.59	47.50 ± 6.38**^/b^	0.34 ± 0.06	375.33 ± 124.53**^/b^	322.50 ± 267.38	2.34 ± 0.55	5.12 ± 0.35
	AgAc 1.55 mg/kg bw^2^	17.42 ± 5.71	51.83 ± 6.24***^/b^	0.34 ± 0.02	214.33 ± 66.45	99.50 ± 83.54	1.36 ± 0.21 ^b^	4.92 ± 0.46

^1^ Nominal dose (actual delivered doses were 0.20 and 0.80 mg/kg bw)

^2^Containing the equivalent dose of 1 mg Ag/kg bw

Statistical significance: ** = *p* < 0.01; ***p < 0.001 vs Control (Kruskal-Wallis test, followed by Dunn’s multiple comparison test); a = *p* < 0.05; b = *p* < 0.01 vs Control (Mann-Whitney U test)

After the recovery period, mice treated with AgNPs showed a dose-dependent decrease of WBCs and lymphocytes. A decrease of lymphocyte number, not associated with a corresponding significant decrease of total WBCs, was recorded in mice treated with AgAc. In mice treated with AgNPs a dose-dependent increase of urea was recorded (associated to a minor increase in albumin at the low dose). Triglycerides increased in a dose-dependent manner compared with controls in groups treated with AgNPs, although this increase was significant only at the highest dosage (1 mg/kg bw). In mice treated with AgAc a significant increase of urea was detected, accompanied by a severe decrease in albumin.

### Silver tissue distribution

**Fig. 3:**
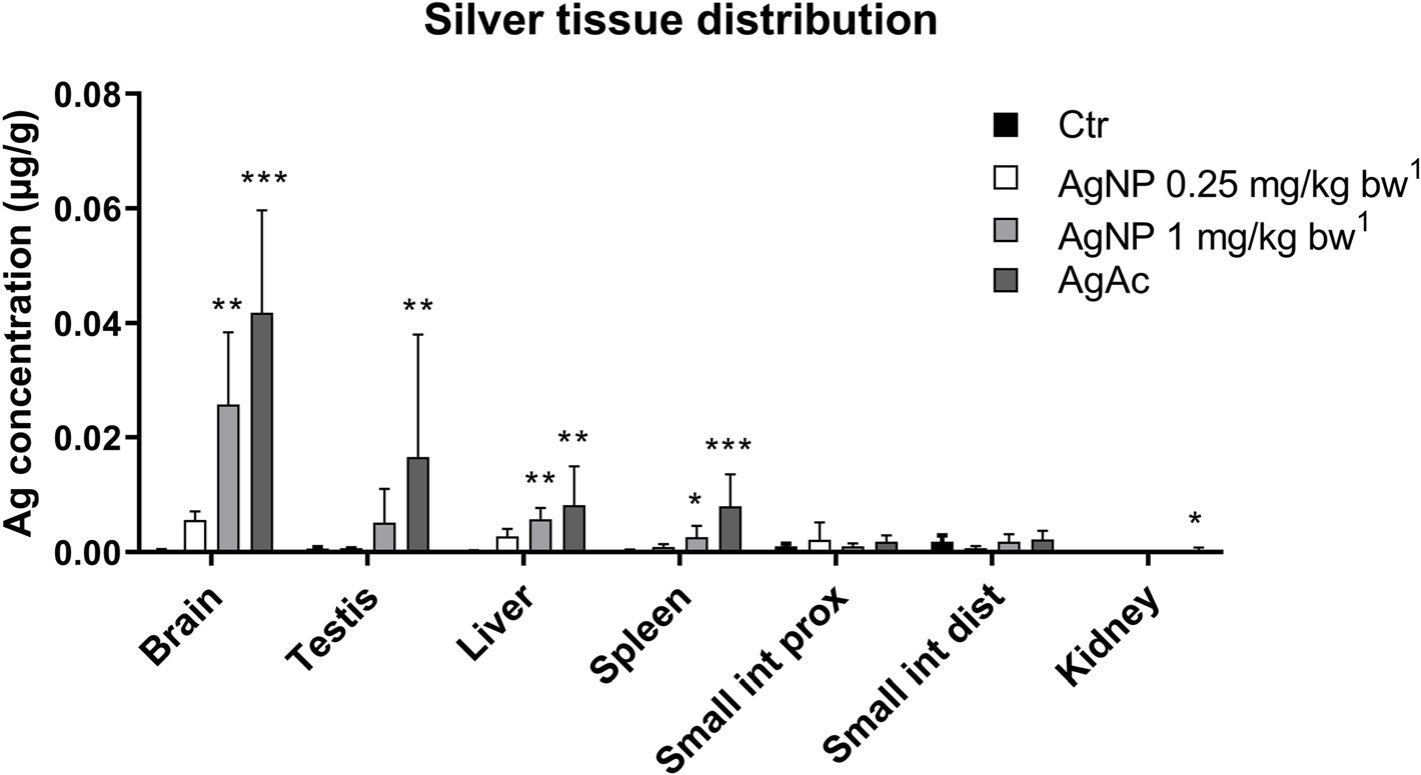
Silver tissue concentration at the end of treatment (EoT) determined by ICP-MS analysis. Data are expressed as mean ± SD. Statistical significance: * = *p* < 0.05; ** = *p* < 0.01; ***p < 0.001 vs Control (Ctr). Kruskal-Wallis test followed by Dunn’s multiple comparison test (*n* = 5). ^1^Nominal dose (actual delivered doses were 0.20 and 0.80 mg/kg bw)

**Fig. 4:**
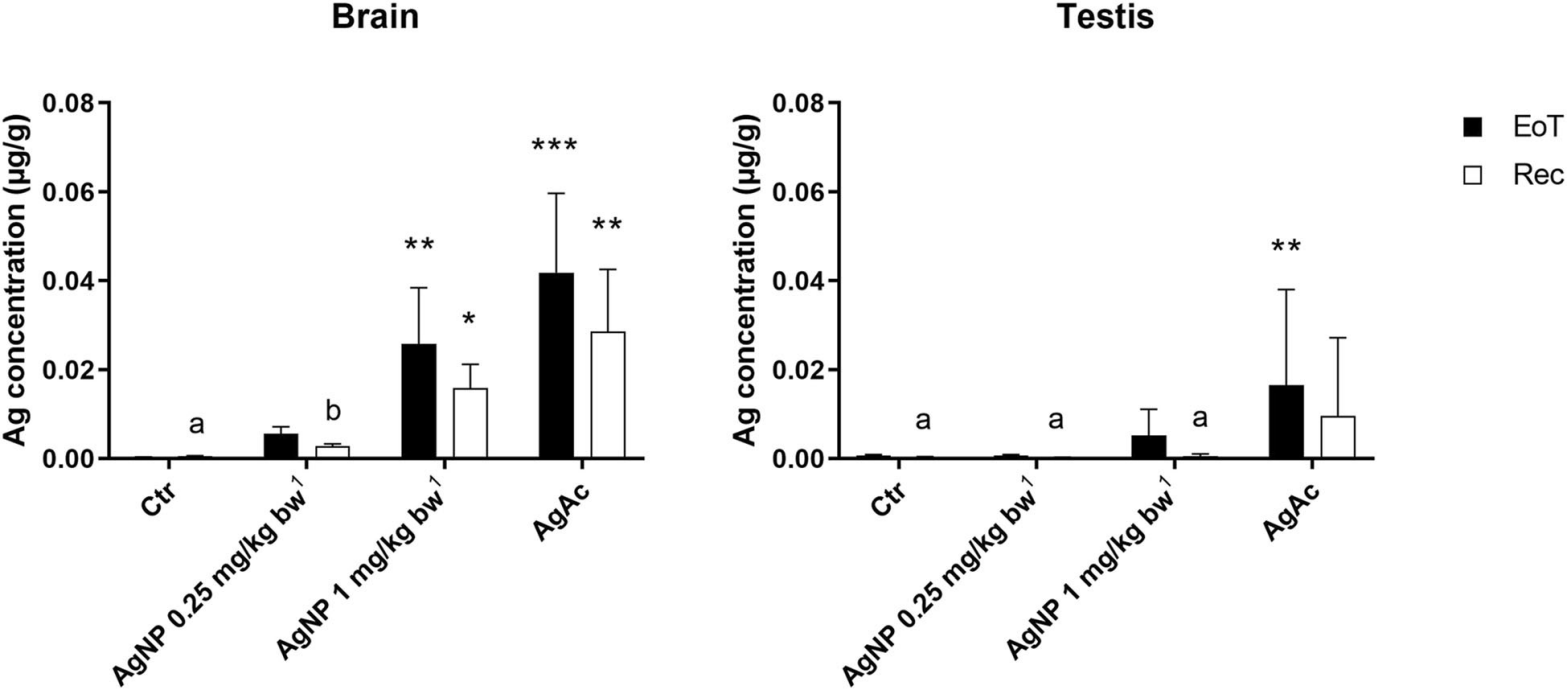
Silver concentration in brain (left) and testis (right) at the end of treatment (EoT) and the recovery (Rec) period determined by ICP-MS. Data are expressed as mean value ± SD. * = *p* < 0.05; ** = *p* < 0.01; ***p < 0.001 vs Ctr (Kruskal-Wallis test, followed by Dunn’s multiple comparison test); a = *p* < 0.05; b = *p* < 0.01 vs EoT (Mann-Whitney test) (*n* = 5). Ctr = control group; ^1^Nominal dose (actual delivered doses were 0.20 and 0.80 mg/kg bw)

At the EoT, the Ag distribution pattern was similar after oral administration of the nano and ionic form of Ag. The highest total Ag concentration was detected in brain, with lower concentrations measured in testis, liver and spleen. Ag was found at a very low concentration in the small intestine (proximal and distal portions) and was scarcely present in the kidneys (Fig. 3). The accumulation of Ag in the organs was dose-dependent after administration of the AgNPs. In mice treated with the high dose of AgNPs, the Ag content in most of the examined organs was slightly lower than in mice treated with AgAc, keeping in mind that the actual delivered dose was 0.8 for AgNPs versus 1 mg/kg bw for AgAc. At the EoT, statistically significant differences in Ag content were found in the brain, liver, and spleen of mice treated with the high dose of AgNPs and in the brain, testis, liver, spleen, and kidney of mice treated with AgAc as compared to control animals. After the recovery period, only a limited decrease of total Ag was observed in brain of mice from all treatment groups (50% in AgNP 0.25 mg/kg bw, 38% in AgNP 1 mg/kg bw, and 31% in AgAc treated mice); deposited Ag was still detectable in the testis as well, although at low concentrations (Fig. 4).

### Histopathology and autometallography

No treatment-related histopathological lesions were identified in the examined organs (liver, spleen, kidney, lung, brain, intestine, testis) of treated animals at the EoT, and after recovery. AMG staining did not reveal any silver accumulation in the examined organs (liver, spleen, kidney, lung, brain, intestine, testis) of animals from all groups at the EoT.

### Immunohistochemistry

Since the brain was found to be the organ with the highest Ag accumulation, in the absence of histopathological changes in H&E-stained sections, immunohistochemical investigations were performed to evaluate specific changes in the cells involved in brain homeostasis and response to injury, such as astrocytes and microglial cells.

**Fig. 5:**
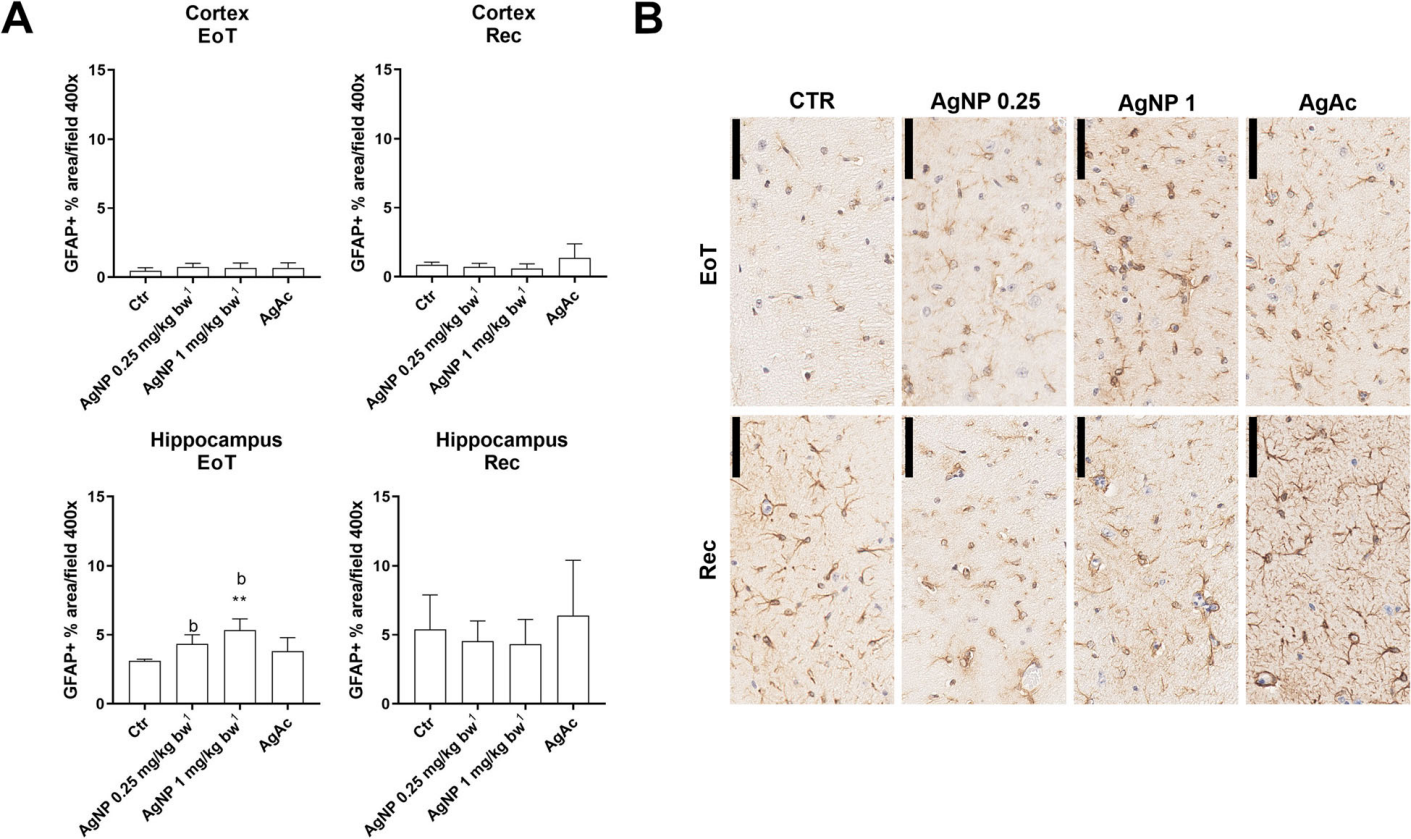
Evaluation of GFAP immunostaining in the brain. **A** Quantification of GFAP (astrocyte) positive % area per field at 400x at the end of treatment (EoT) and recovery (Rec) in the frontal cortex and hippocampus. Data are expressed as means ± SD. Statistical significance: ** = *p* < 0.01 vs Ctr (Kruskal-Wallis test, followed by Dunn’s multiple comparison test); b = *p* < 0.01 vs Ctr (Mann-Whitney test) (*n* = 5). Ctr = control group; ^1^Nominal dose (actual delivered doses were 0.20 and 0.80 mg/kg bw). **B** Representative images of GFAP immunostained sections of hippocampus (stratum lacunosum-moleculare), scale bar = 50 μm

An increase of GFAP-positive % area (marker of astrocytes) was observed in the hippocampus (but not in the cortex) at the EoT in AgNP treated mice as compared to control mice, while after recovery no differences could be noticed (Fig. 5).

**Fig. 6:**
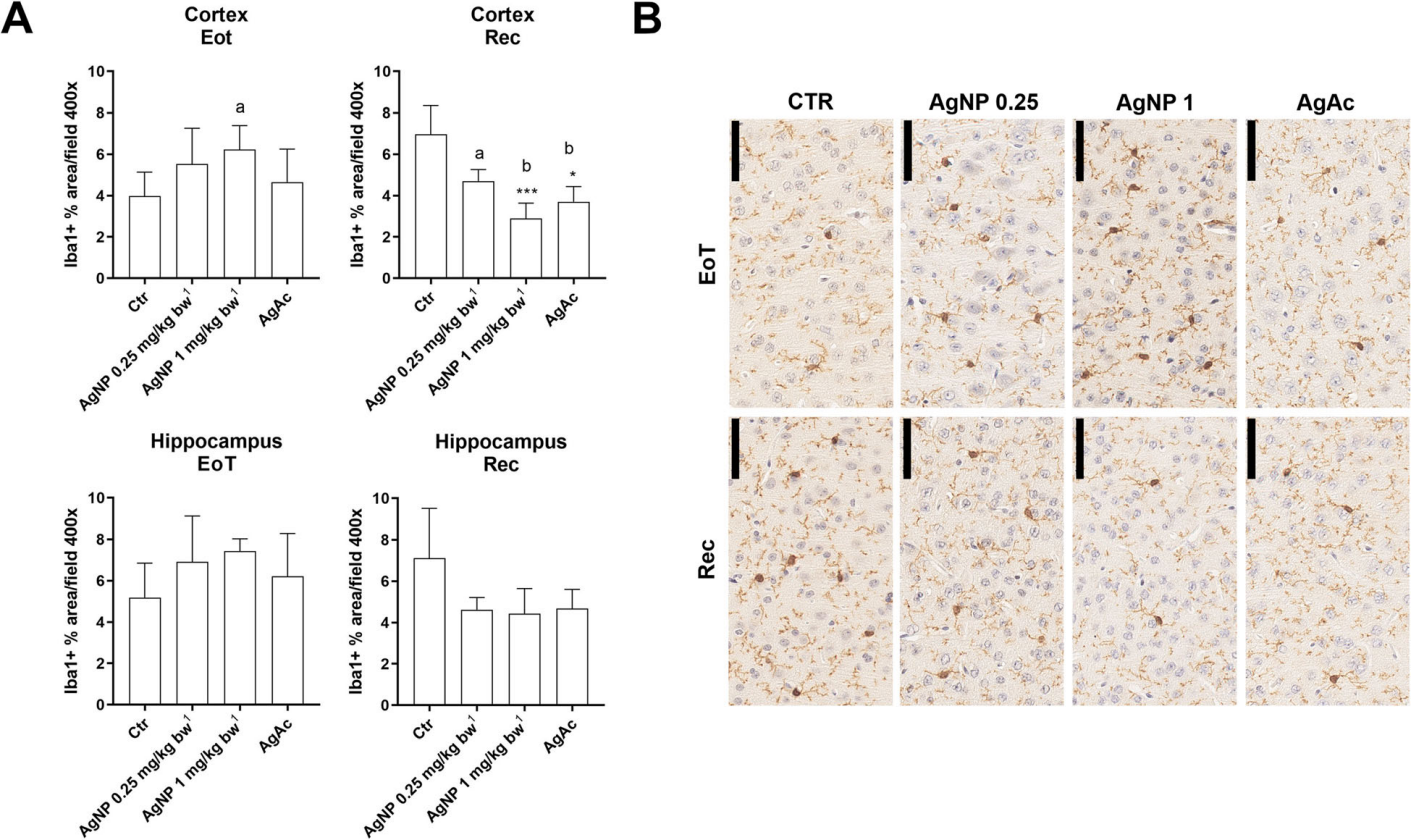
Evaluation of Iba1 immunostaining in the brain. **A** Quantification of Iba1 (microglia) positive % area per field at 400x at the end of treatment (EoT) and recovery (Rec) in the frontal cortex and hippocampus. Data are expressed as means ± SD. Statistical significance: * = *p* < 0.05; ***p < 0.001 vs Ctr (Kruskal-Wallis test, followed by Dunn’s multiple comparison test); a = *p* < 0.05; b = *p* < 0.01 vs Ctr (Mann-Whitney test) (*n* = 5). Ctr = control group. ^1^ Nominal dose (actual delivered doses were 0.20 and 0.80 mg/kg bw). **B** Representative images of Iba1 immunostained sections of frontal cortex, scale bar = 50 μm

An increase of the Iba1-positive % area (marker of microglia) was observed at the EoT in the cortex and hippocampus of all treated animals, significant only in the cortex of AgNP treated mice at the high dose. After the recovery period there was a decrease of Iba1-positive % area in all treated animals as compared to control mice, significant only in the cortex of AgNP treated mice at the high dose (Fig. 6).

Immunohistochemistry for albumin in brain sections was performed to detect potential BBB damage, but immunohistochemical staining did not reveal any signs of albumin extravasation from brain microvasculature in either control or treated mice at the EoT (Fig. S1).

### TEM analysis

**Fig. 7:**
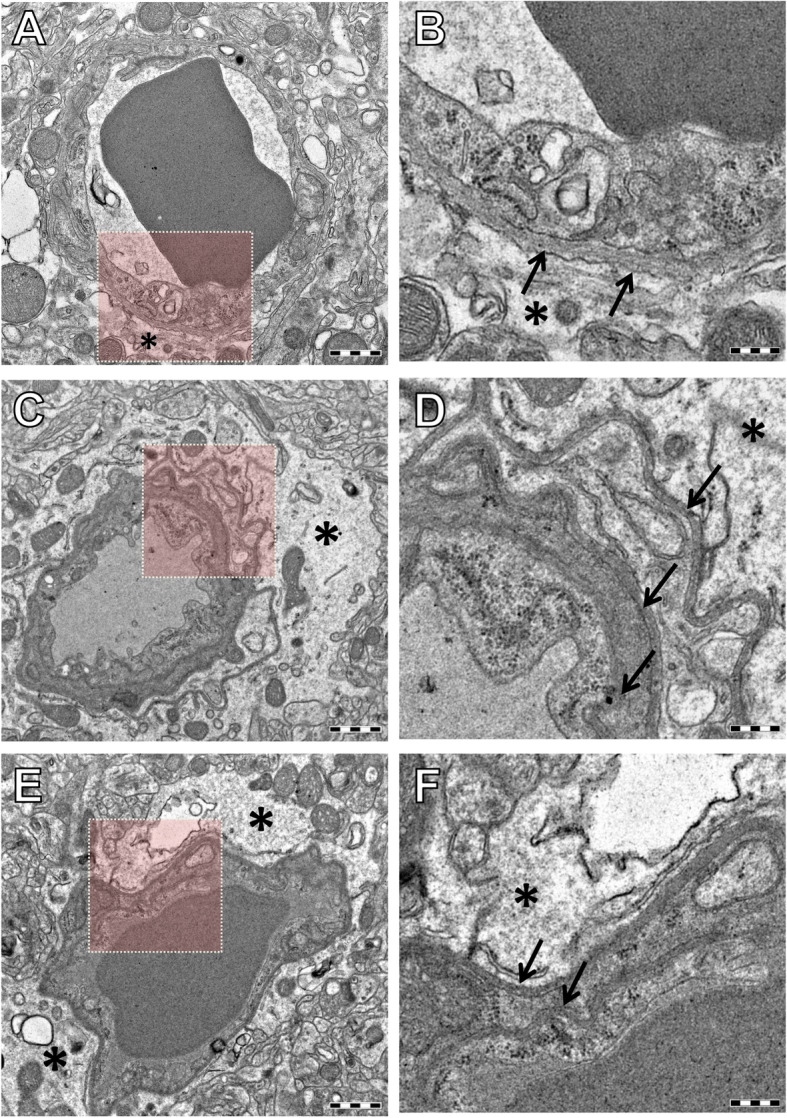
Representative TEM images of capillaries in the hippocampus of mice at the end of treatment. **A**-**B** control mouse; **C**-**D** AgNP 1 mg/kg bw-treated mouse; **E**-**F** AgAc-treated mouse. **A** Normal capillary and astrocytic perivascular end-feet (asterisk) from a control mouse; **B** higher magnification of the area highlighted in A showing a portion of normal blood-brain barrier (BBB) with basement membrane (black arrows), and astrocytic perivascular end-feet (asterisk). Swelling of astrocytic perivascular end-feet (asterisk) was observed in AgNP 1 mg/kg bw- (**C**-**D**) and AgAc- (**E**-**F**) treated mice. Splitting of capillary basement membrane in three and two different branches (black arrows) visible at higher magnification of the BBB, respectively in AgNP 1 mg/kg bw- (**D**) and AgAc- (**F**) treated mice. Scale bar = 1µm in panel **A**, **C**, **E**; scale bar = 300 nm in panel **B**, **D**, **F**

TEM analysis of the hippocampus revealed splitting of basement membrane of the capillaries and swelling of astrocytic perivascular end-feet in both AgNP (high dose)- and AgAc-treated mice (Fig. 7), while no changes were found in the control mouse. No ultrastructural changes were identified at level of the cortex. At the end of the recovery period, mild residual splitting of basement membrane of the capillaries was observed only in the hippocampus of the AgAc-treated mouse (data not shown). No morphological abnormalities were detected in myelin sheaths or synaptic structures (Fig. S2).

TEM analysis of the small intestine of control and treated mice revealed a normal ultrastructure. Silver deposits were not detected in the examined areas of hippocampus and small intestine.

## Discussion

The increasing application of AgNPs in food-related areas and in many consumer products raises concerns about their potential risk to human health. Although some studies suggested that the toxic effects after exposure to AgNPs by different routes were overall mild, available evidence is limited and heterogeneous owing to differences in study design and in the physicochemical properties of the test materials. The opinion expressed by the SCENIHR (2014) highlighted that data are insufficient to carry out a full risk assessment as information on possible long-term effects are lacking and more studies on health effects after long term exposure are needed. Recent studies drew attention to effects of AgNPs on CNS as a target organ for Ag accumulation and toxicity [33–44].

In a previous study we demonstrated that exposure to 10 nm AgNPs resulted in a greater tissue distribution and induced more toxic effects than larger AgNPs (40 and 100 nm) after single intravenous administration in mice [45]. Since low-level oral exposure is a relevant route of exposure to nano-silver, the current study was conducted to assess the biodistribution and toxicity of 10 nm AgNPs after repeated oral administration in mice at pertinent doses, based on the expected human exposure, and compare their effects with those induced by an almost equivalent dose of Ag in the ionic form (AgAc).

The magnitude of the aggregated oral exposure to AgNPs can only be roughly estimated. The EFSA ANS Panel assessed the exposure to the food additive E174 (maximum level scenario) to be up to 12.0, 8.6, 3.6 μg/kg bw/day for children (3–9 y), adolescents (10–17 y), and adults (18–64 y), respectively [49]. The mass fraction of nanoparticles based on De Vos et al. (2020) is 0.17–0.55% in pristine E174 and 0.16–0.53% in E174-containing confectionery [46]. However, exposure to E174 is likely to be a minor part of the total oral exposure when all sources are considered, including unintentional ingestion via handling of consumer products incorporating AgNP, especially in children. For instance, Bachler et al. (2013) put forward different exposure scenarios for validating their PBPK model, including diet (90 μg/day), release from a commercial food box (4.2 μg/day) or use of a throat spray (modeled for inhalation exposure there, but potentially leading to oral exposure as well) [50]. Other realistic scenarios contributing to the aggregated oral exposure include use of Ag NP-containing toothpaste and inadvertent ingestion resulting to wearing/handling Ag NP-containing dresses, textiles and other surface treated consumers products. In the present study, the administered doses of 1 and 0.25 mg/kg bw in mice are a factor 100 and 25 higher than the human exposure of roughly 10 μg/kg bw/day from diet alone, which cover the common safety factor of 100 for intraspecies and interspecies differences [51].

Since AgNPs are known to degrade over time [47], a thorough characterization of AgNPs before and during the experiments was undertaken. TEM analysis confirmed that the size (10 nm) and shape (spherical) of AgNPs were the intended ones, whereas characterization of AgNPs aliquots before and after use revealed that AgNPs did not substantially change their dimension and aggregation status over time and therefore were suitable for the in vivo experiments. The quantitative determination of the total Ag concentration in the AgNP batch used in the experiments was performed at the end of the dosing period, when the quantification of the Ag tissue contents became available and raised doubts on the Ag mass concentration declared by the manufacturer. Actual measurement revealed that the Ag concentration of the AgNP batch was 80% of that stated by the manufacturer, highlighting the importance of not relying on declared values accompanying commercial batches for mass concentrations, as usually done in the large majority of studies, and not only for physical properties such as size and shape.

The biodistribution study revealed that detectable Ag concentrations were present in all examined organs albeit at relatively low levels, as expected on the basis of the low doses administered. Even considering that the actual delivered dose of silver was 0.8 mg/kg bw for AgNPs versus 1 mg/kg bw for AgAc, in all examined organs (except the intestine) the highest silver concentrations were found in AgAc treated animals, supporting the evidence that Ag ions are able to pass the gastro-intestinal membrane and reach target organs more than AgNPs, as previously reported [26, 27].

In previous oral studies performed in rodents where silver (ionic and nano-form, different sizes) was administered with variable exposure durations and intensities, silver was reported to distribute in a wide range of organs, including the gastrointestinal tract, liver, spleen, testis, kidney, brain, and lungs [25–28, 30]. In our study proportionally lower Ag concentrations were found in the intestinal tract compared to the results obtained by other authors after oral administration of 14–20 nm AgNPs and ionic silver (AgAc or AgNO_3_) for 28 days at 9 mg/kg bw, where the intestinal tract exhibited the highest Ag concentrations [26, 27]. It is to be noted that in our study mice were sacrificed 3 days after the last administration and since 24 h after oral ingestion about 60 and 40% of administered AgNPs and AgAc, respectively, can be excreted in feces [26] it is possible that residual Ag-containing intestinal content affected the measurement of tissue levels in those earlier studies. It is also worth noticing that due to the quick enterocyte turnover (< 3 days) [52], the detected concentrations in the present study highlight the potential for Ag accumulation in the intestine with long-term exposure, even at low levels.

The observed accumulation of silver in the brain (and to a lesser extent, in testis) and the limited decrease observed (ca. 30–40% at 1 mg Ag/kg bw exposure levels) after an elimination phase of 4 weeks were of concern. The Ag concentrations detected in the brain and testis of AgNPs and AgAc treated animals on the one hand confirm the capability of Ag (either as AgNPs or Ag ions) to cross the blood brain and the ematotesticular barriers, and on the other hand can be related to limited clearance capacity of these organs leading to progressive accumulation [27, 28, 53]. After recovery, Ag was still present in the brain and testis at significant levels, indicating slow clearance and remarkable persistence of Ag in these organs [27, 28]. In case of daily exposure, this behavior may result in Ag accumulation over time.

AMG staining and TEM imaging (performed only on intestine and brain) failed to identify silver particles or agglomerates in the examined tissues and their exact localization at tissue/cellular level. This outcome was indeed expected on the basis of the low exposure levels and the very low concentrations of silver detected in the organs (range 0.001–0.07 μg/g), since the positive detection by AMG was associated with tissue levels ≥3 μg Ag/g [45]. Although we were not able to detect silver particles in the brain, previous oral studies performed in rats using similar doses (0.2 mg/kg bw) and particles (10 nm) identified silver nanosized granules in the brain by TEM [39, 43], confirming their ability to cross the BBB.

As regards to effects induced by low-dose oral administration of silver in this study, it is noted that in the absence of treatment-related clinical signs, mortality, and statistically significant effects on body and organ weights at the EoT and after recovery, AgNP treated animals at the low dose showed an increased body weight and body weight gain at the EoT (although not statistically significant), similarly to what reported in rats orally treated with 10 nm AgNP for 14 days at the dose of 0.2 mg/kg [40]. This might suggest a potential effect of low doses of AgNPs on growth performances, as found in pigs treated with metallic silver [54], which could be mediated by Ag antimicrobial properties (as reported for antibiotic growth promoters in livestock) [54, 55]; however, the exact mechanisms and the long-term consequences of such low dose-level exposure, as highlighted by the present study, should be carefully considered.

Hematological and clinical chemistry analyses revealed significant treatment-related changes. At the EoT, no significant changes were observed in hematological parameters except for a slight increase of MHC in AgAc-treated mice that is likely not biologically relevant, since MCHC (the other indicator of intraerythrocytic haemoglobin) did not significantly increase and changes of MCH and MCHC are considered pathophysiologically relevant when anemia is present, which was not the case [56, 57]. Clinical chemistry revealed a decrease of urea in mice treated with the low dose of AgNPs that could theoretically be associated with decreased liver function, that in turn may also lead to a decreased activity of hepatic enzymes such as GLDH and ALT. However, a decrease of enzyme activity is considered not relevant by several laboratories or textbooks [58]. Additionally, a decreased serum enzyme activity may be consistent with liver failure only if the latter depends on a severe reduction of the hepatic mass, which was not noticed at necropsy or histologically and that would have been associated with a severe decrease of albumin and/or with a severe increase of triglycerides due to a decreased hepatic metabolization of lipids [59, 60], not detected in the current study. Moreover, the same changes were not recorded at the highest dosage of AgNP, suggesting that at this dosage, liver failure can be confidently excluded. Ultimately, clinical chemistry revealed no treatment-related and dose-dependent organ damage or dysfunction at the EoT.

After the recovery period, lymphopenia was observed in mice treated with both AgNPs, and AgAc. However, considering the delivered doses, lymphopenia was much more severe in AgNP treated mice and in that case only it was accompanied by a dose-dependent decrease of total WBCs. Immunotoxic effects were previously reported after repeated intravenous administration for 28 days of 20 and 100 nm AgNP up to a maximum dose of 6 mg/kg bw: several immune parameters were affected, such as thymus weight (reduced), spleen weight and spleen cell number (increased), NK cell activity (strongly reduced), IFN-γ production (reduced), and T cell dependent antibody response (suppressed) [61, 62]. The design of our study does not allow to understand whether the observed WBC decrease depended on a decreased lymphocyte blastogenesis in lymphoid organs or to an increased peripheral consumption of lymphocytes [56]. The only examined lymphoid organ, i.e. the spleen, had no changes in absolute weight and histologically lymphoid depletion was not observed. However, given that lymphopenia was detected with both AgNP and AgAc treatments, a direct effect of silver on lymphocytes is apparent. On the other hand, the fact that in mice treated with AgNPs this adverse effect was more severe and dose-dependent, does show the existence of an underlying nano-specific mechanism and indirectly support the presence of AgNPs in the systemic circulation or in the lymphoid organs. Further evidence in this respect is offered by the triglyceride increase seen in AgNP-treated mice only, which again was dose-dependent and significant at the high dose. It is worth noticing that ALT was markedly increased in AgNP-treated mice at the high dose as well (although the increase was not statistically significant). Differently, the urea increase was seen in all treatment groups and was broadly dose dependent, irrespectively of the nature of the administered silver (AgNPs or ionic).

Albumin changes were observed in mice treated with AgNP at the low dose (increased) and AgAc (decreased). The slight increase of albumin might be associated with dehydration, which in turn might also explain the urea increase in the absence of hypercreatininemia and the slight (not significant) increase in the hematocrit [63]. The increase of triglycerides in AgNP-treated mice in the absence of other signs of liver failure or toxicity may depend on an increased food intake or lipomobilization [59]. However, no changes in body weight or other signs of lipomobilization (e.g., fatty liver) were found. Hence, future studies on the possible mechanisms responsible of hypertriglyceridemia in mice treated with AgNP and its potential long-term effects are warranted.

The severe decrease of albumin after recovery in mice treated with AgAc may be considered truly pathological and is another evidence clearly distinguishing AgNPs and Ag-ionic related effects. Among the possible causes of hypoalbuminemia, liver failure or severe inflammation may be excluded based on other hematological or biochemical results. Decreased food intake has not been observed and is not consistent with body weight data. Similarly, gastrointestinal signs or lesions potentially responsible for intestinal protein loss have not been observed in this study. The only possible explanation of this finding relies thus in a possible renal loss of proteins [64]. Unfortunately, urine samples were not collected to measure proteinuria and no renal tissue samples were collected for ultrastructural investigation of the glomerular structure. However, since AgAc is excreted through the kidney, differently from AgNPs that appear to be mainly excreted through the liver [45, 65, 66], the possibility of damage in the renal filtrating system due to the excretion of ionic silver over time can be reasonably put forward. This hypothesis is further supported by the limited release of silver ions from 10 nm AgNPs in the serum, since the percentage of ionic silver at 24 h was 0.005% [45].

No treatment-related histopathological lesions were identified in any group of treatment. Our results are consistent with previous studies reporting no or incospicous effects on host tissues caused by repeated oral administration of AgNP [24, 25, 28, 29, 67] and were expected given the low doses administered.

A particular case in the present study is that of the brain, the organ with the highest Ag accumulation in both AgNP- and AgAc-treated groups. Therefore, we focused our investigation on this organ even though no morphological changes were histologically evident. By using immunohistochemistry, we investigated the effects of treatment on glial cells as the main cells involved in brain response to injury and in the protection of the brain against oxidative stress and metal toxicity [68, 69]. In doing so, we considered that interaction between microglia and astrocytes in vivo may be an important element in the evolution of an inflammatory pathology [70]. At the EoT, in mice treated with AgNPs, we found an increase of immunoreactivity area of GFAP+ astrocytes in the hippocampus (more evident at the high dose), and an increase of immunoreactivity area of Iba1+ microglial cells both in the hippocampus and cortex, although this increase was significant only in the cortex of mice treated with AgNPs at the high dose. These results suggest that glial cells were activated following treatment with AgNPs (but not AgAc), and that this effect was dose dependent. At the end of the recovery period, on the contrary, we observed a decrease in the Iba1 immunoreactivity area in all treated groups, with no effects on GFAP+ astrocytes, indicating that the activation of glial cells was reversible. The decrease of microglial immunoreactivity area after a 4-week recovery period could correspond with the resolution phase of microglial activation as reported in mice treated with lipopolysaccharide (LPS) as an experimental model of glial activation [71]. Further confirmation of astrocyte involvement was demonstrated by TEM analysis of hippocampus that revealed swelling of astrocytic perivascular end-feet in both AgNPs (high dose) and AgAc treated mice. Similar morphological effects were reported in other studies after administration of AgNPs by different routes [35,36,44,]. In addition to ultrastructural changes of astrocytes, also splitting of capillary basement membrane was evident in both AgNPs (high dose) and AgAc treated mice and still detectable at the end of the recovery in AgAc treated mice. Whereas these ultrastructural changes were similar in mice treated with nano and ionic form of silver, activation of glial cells at the EoT following exposure to AgNPs, but not AgAc, suggests a nano-specific mode of action of silver in the brain.

In the present study, no morphological abnormalities in myelin sheaths or synaptic structures were identified, differently from earlier reports after oral administration in rats of 10 nm AgNPs and AgAc at the dose of 0.2 mg/kg bw [39, 40]. Altogether our findings indicate that silver induced a morphological change of the BBB, but immunohistochemical staining of brain sections for albumin did not reveal any signs of albumin extravasation from brain microvasculature at EoT, indicating that the magnitude of BBB damage detected was eventually mild. However, in the light of the potential for accumulation upon long-term exposure, the effects seen in the brain in the present study are of concern. In particular, activation of glial cells observed upon AgNP exposure was reversible in the conditions of this study, i.e. after short-term exposure, but might not be such when the exposure is continuous and AgNPs can indeed accumulate to higher levels. Glial activation associated with neuroinflammation is considered one of the mechanisms involved in the onset of mental disorders and the role of metal-based nanoparticles as potential triggering agents is currently debated [72–74].

## Conclusions

Four-week low-dose oral administration of silver as AgNP and AgAc in mice induced subtle but well-defined changes which highlight issues of concern to be addressed by further research. Part of these changes were associated with exposure to AgNPs, part to ionic Ag, and others to both Ag forms. Ag accumulation in brain and testis as well as increased urea concentrations were apparent after treatment with both Ag forms. Lymphopenia and increased triglycerides were hallmarks of AgNP treatment. Decreased albumin was observed after AgAc treatment only. In the brain, activation of glial cells was mainly evident after AgNP treatment, while ultrastructural changes of the BBB were observed after treatment with both Ag forms. Central nervous system effects, along with the slow clearance of silver from this organ, deserve attention and should be considered in the risk assessment of AgNPs.

## Methods

### Physicochemical characterization of silver nanoparticles

Suspensions of BioPure™ Silver Nanoparticles (AgNPs) of 10 nm in size coated with citrate (CT-AgNPs, batch no. DAG2289) were purchased from NanoComposix (San Diego, USA) at the concentration of 1.0 mg/mL. BioPure™AgNPs were selected because they are sterile and with an endotoxin level ≤ 2.5 EU/mL. To avoid any contamination during the dosing period, the AgNPs were divided in 4 aliquots (A, B, C, D) immediately after delivery, then they were stored at + 4 °C, according to manufacturer’s instructions. Before characterization, AgNPs were diluted with 2.0 mM sodium citrate (cod. W302600, Sigma-Aldrich) buffer. Samples were sonicated in a ultrasonic unit (Elmasonic S 30 H) for 30 s in accordance with the manufacturer’s instructions. To prevent contamination, measurements were run using disposable plastic cuvettes. The quality of the dispersions was checked before the beginning of each dosing week (at day − 3, 4, 11 and 18) by Dynamic Light Scattering (DLS) and UV-visible (UV-vis) spectroscopy. At the end of each dosing week (at day 4, 11, 18 and 25) the leftover was further analysed by UV-Vis spectroscopy (Fig. 2).

#### Transmission Electron Microscopy (TEM)

Formvar coated copper grids (cod. PE1GC300, Pelco) were pre-treated with 20 μl of poly-L-lysine 0.01% (w/v) (Sigma Aldrich) for 15 min. After washing twice with MilliQ water, 3 μL of AgNPs suspensions were deposited onto the grid for 5 min and then rinsed with 3 μL of 2-propanol (Sigma Aldrich). According to the manufacturer’s advice, 10 nm AgNPs were diluted up to 0.1 mg/mL before use. The grids were allowed to dry overnight at room temperature in a covered crystallizing dish. Images were taken with a FEI Tecnai G2 (Eindhoven) and analyzed with the ImageJ software (http://imagej.nih.gov/ij/). Feret diameter was determined to assess the particle size.

#### Dynamic Light Scattering (DLS)

The hydrodynamic diameter of AgNPs in dispersion was measured with a Malvern Zetasizer Nano ZS90 operating with a light source wavelength of 633 nm and a fixed scattering angle of 90°. In order to optimize the scattering intensity, the AgNPs were diluted 1:10 in sodium citrate 2.0 mM. All measurements were run at room temperature (RT) for at least three times.

#### UV-Visible (UV-Vis) spectrophotometry

The UV-Vis spectra were acquired in the 300–700 nm range using an Agilent Cary 100 Spectrophotometer. Due to the high adsorbance of the naporaticles, AgNPs were diluted 1:200 in sodium citrate 2.0 mM. All measurements were run at RT for at least three times, on three different replicates.

#### Ag mass concentration

The total Ag concentration of the suspensions was determined by triple quadrupole inductively coupled plasma mass spectrometry (ICP-MS). Suspensions were sonicated with an ultrasonic system (USC900TH, VWR International) for 30 s, diluted with 0.1% HNO_3_ and analysed in triplicate as described below for tissues.

### Animals and experimental design

Male CD-1(ICR) mice aged 4–5 weeks at arrival were purchased from Charles River (Calco, Italy). They were housed in standard Individually Ventilated Cages (IVC; GM500, Tecniplast, Buguggiate, Italy) and acclimated to the environment for a week prior to the initiation of the study, with free access to water and a standard pellet diet ad libitum. The environmental conditions were set at a temperature of 22 ± 2 °C, relative humidity of 55 ± 10% and a 12 h light/dark cycle.

Mice were randomly divided into control and exposed groups of treatments (*n* = 6 animals per group). Mice received 10 nm CT-coated AgNPs by oral gavage at two dose levels [0.25 and 1 mg/kg body weight (bw)]. For comparison, a group was treated with silver acetate (AgAc), used as source of Ag ions, at a dose of 1.55 mg/kg bw, containing the equivalent dose of 1 mg Ag/kg bw. The control group was treated with sterile water. The dosing volume was 10 ml/kg bw. Mice received the treatment by oral gavage as repeated administration for 4 weeks (once a day for 5 days/week). A group of mice was sacrificed 3 days after the end of treatment (EoT) and a group of animals was monitored and sacrificed at the end of the recovery period (Rec) (28 days after the EoT) (Fig. 2). The body weight of each mouse was measured every day before the treatment during the dosing period, twice a week during the recovery period, and at sacrifice. Mice were euthanized by carbon dioxide inhalation using a gradual 20% vol/min displacement rate [75].

Animals were maintained according to the guidelines set out in Commission Recommendation 2007/526/EC of 18 June 2007, for the accommodation and care of animals used for experimental and other scientific purposes and were used in accordance with the Italian laws (D.L 26/2014) enforcing the Council Directive 2010/63/UE. The experiment was approved by the Italian Ministry of Health (approval no. 942/2015-PR, issued on 04 September 2015).

### Sampling

At sacrifice, blood was drawn from the heart and immediately placed in tubes containing EDTA, stored at room temperature and transported to the laboratory for hematology and clinical chemistry analyses. Mice underwent complete necropsy, and the weight of liver, spleen and kidneys was measured. Liver, spleen, kidneys, lung, testes, brain, and intestine were collected for silver quantification and histopathological examination. Brain (hippocampus, cortex) and small intestine were additionally collected for transmission electron microscopy (TEM) analysis. For quantification of Ag a portion of the collected organs was stored at − 80 °C pending analysis.

### Hematology and clinical chemistry

Blood cell count was performed using a laser-based counter (Sysmex-XT 2000iV) with a species-specific software. A blood smear was prepared, samples were centrifuged, and plasma (300 to 750 μLs) was transferred in another tube and frozen at − 20°. WBC differential, cell morphology, and platelet estimate were determined on May Grünwald Giemsa stained smears. Clinical chemistry was performed on thawed plasma with an automated spectrophotometer (ILAB-300, Instrumentation Lab). Specifically, the activity of Glutamate dehydrogenase (GLDH) and alanine aminotransferase (ALT) and the concentration of Urea, Creatinine, Triglycerides, Albumin and Total protein were measured using reagents provided by the manufacturer of the instrument.

### Quantification of Ag in tissues

Total Ag content was determined in whole blood and in organs - liver, spleen, kidneys, small intestine (proximal and distal tracts), brain, and testes - by means of triple quadrupole ICP-MS. A 8800 ICPQQQ spectrometer (Agilent Technologies, Japan, Tokio) equipped with an autosampler, a peristaltic pump, a Micro-Mist glass concentric nebuliser, and operated at a RF power of 1550 W, was used. All sample manipulations were carried out in clean room conditions under a laminar flow box. Samples were placed in high-pressure Teflon containers with 3 mL of HNO_3_, 0.5 mL of H_2_O_2_ (both ultrapure grade, Carlo Erba, Rodano, Italy), and digested with a microwave system (UltraWAVE Single Reaction Chamber Microwave Digestion System, Milestone, Bergamo, Italy). The irradiation program consisted in 23 min at 220 °C (ramp), 10 min at 220 °C (hold, maximum power 1400 W), 15 min depressurization and cooling at room temperature. Intestine samples were previously submitted to an accurate cleaning procedure developed in earlier studies to avoid contamination from gastrointestinal digestion residues [76]. After cooling, the digests were diluted by adding HCl (final concentration 3.0 M) to promote the formation of soluble silver complexes and prevent the precipitation of insoluble Ag + salts. Prior to analysis the digests were highly diluted with 0.1% HNO_3_ and the appropriate amount of HCl so as to maintain silver in complexed form. Measurements were carried out on ^107^Ag and ^103^Rh, as internal standard by the method of external calibration. The method detection limit ranged from 0.4 to 0.7 μg/kg tissue, depending on the tissue, and was 0.09 μg/l for blood. Trueness was assessed by analyzing the certified reference material SRM 1577c Bovine Liver (NIST, Gaithersburg, MD, USA), with a certified value for silver of 5.9 ± 1.6 μg/kg and the control material Seronorm™ Trace Elements Whole Blood L-1 (SERO AS, Billingstad, Norway) with an indicative value for silver of 185 ± 10 ng/l, both included in every analytical batch. The average determined silver concentrations were 6.0 ± 0.5 μg/kg (*n* = 6) and 179 ± 2 ng/l (*n* = 6) for the liver-based and the blood-based material, respectively. The trueness of determinations was also assessed through spikes of known amounts of silver in tissues and blood before sample dissolution, giving recoveries within the range of 90–100%, with no appreciable differences between sample types.

### Histological and histochemical examination

For histological examination, liver (median lobe including the gall bladder), spleen (apical portion), kidney (half of the right kidney), lung (left lobe), brain (coronal sections obtained by using Adult Mouse Brain Slicer Matrix BSMAS005–1, Zivic Instruments, USA), small intestine (duodenum, jejunum, ileum), and large intestine (colon, caecum), were fixed in 10% neutral buffered formalin for at least 48 h at room temperature, routinely processed for paraffin embedding, sectioned at 4 μm thickness, stained with hematoxylin-eosin (H&E, Mayer’s haematoxylin, cat. No. C0302; Eosin G, cat. No. C0362, Diapath, Martinengo, Bergamo, Italy), and evaluated under a light microscope. Outcome assessors were blinded to the study groups.

To analyze the tissue distribution and localization of silver, autometallography (AMG) [77] was performed on serial sections. After AMG staining, sections were counterstained with safranin O and evaluated under a light microscope for the identification of tissue and cellular localization of silver, visible as black granular pigment.

### Immunohistochemistry

For immunohistochemistry (IHC), brain sections were immunostained with the following primary antibodies: rabbit polyclonal anti-IBA1 (marker of microglia, Wako Chemicals, Richmond, VA, USA, cat. No. 019–19,741), rabbit polyclonal anti-GFAP (marker of astrocytes, Dako, Glostrup, Denmark, cat. No. Z0334), and rabbit polyclonal anti-albumin (to detect blood brain barrier damage, Abcam, Cambridge, UK, cat. No. ab19196). Sections were then incubated with biotinylated secondary antibody (goat anti-rabbit, Vector Laboratories, USA, cat. No. VC-BA-1000-MM15), labelled by the avidin-biotin-peroxidase procedure (VECTASTAIN® Elite ABC-Peroxidase Kit Standard, Vector Laboratories, USA, cat. No. VC-PK-6100-KI01). The immunoreaction was visualized with 3,3′-diaminobenzidine (DAB, Peroxidase DAB Substrate Kit, Vector Laboratories, USA, cat. No. VC-SK-4100-KI01) substrate and sections were counterstained with Mayer’s haematoxylin (Diapath, Martinengo (BG), Italy, cat. No. C0302). Outcome assessors were blinded to the study groups.

### Digital image analysis

Iba1- and GFAP-positive % areas were measured in 4400x microscopic fields using the ImageJ analysis program (http://rsb.info.nih.gov/ij/). Brain areas analysed were selected in the frontal cortex and the hippocampus (specifically the CA1- CA2 and CA3 regions). Outcome assessors were blinded to the study groups.

### Transmission electron microscopy

Brain (hippocampus and cortex) and small intestine samples were reduced and fixed with 4% paraformaldehyde (PFA) and 2% glutaraldehyde in phosphate buffer 0.12 mol/l pH 7.4 for 6 h, followed by incubation at room temperature for 2 h in 1% OsO_4_. After dehydration in a graded series of ethanol preparations, tissue samples were cleared in propylene oxide, embedded in epoxy medium (Epoxy Embedding Medium kit; Sigma-Aldrich, St. Louis, MO 63103 USA) and polymerised at 60 °C for 72 h. From each sample, one semi-thin (1 μm) section was cut with a Leica EM UC6 ultramicrotome (Leica Microsystems, Vienna, Austria), stained with Toluidine Blue and mounted on glass slides to identify the areas of interest. Ultra-thin (60 nm thick) sections were then obtained, counterstained with uranyl acetate and lead citrate, and examined with an energy filter transmission electron microscope (Libra120, Carl Zeiss NTS GmbH, Oberkochen, Germany) equipped with a yttrium aluminium garnet (YAG) scintillator slow-scan charge-coupled device (CCD) camera (Sharp eye, TRS, Moorenweis, Germany).

### Statistical analysis

Data were analyzed using Graph Pad Prism version 8.0 (GraphPad Software, San Diego, CA). Nonparametric tests (i.e., Kruskal-Wallis test followed by Dunn’s multiple comparison test, Mann-Whitney U test) were used to detect differences among groups. The *P*-values < 0.05 were considered statistically significant.

## Supplementary Information

**Additional file 1: Figure S1.** Brain, hippocampus, immunohistochemistry for albumin.

**Additional file 2: Figure S2.** Representative TEM images of hippocampal synapses and myelin sheaths at the end of treatment.

## Data Availability

The datasets generated in this study are available from the corresponding author on reasonable request.

## Abbreviations

AgAc: Silver acetate
AgNP: Silver nanoparticle
AMG: Autometallography
bw: Body weight
DLS: Dynamic light scattering
EoT: End of treatment
H&E: Hematoxylin and eosin
ICP-MS: Inductively coupled plasma mass spectroscopy
PO: Per os
Rec: Recovery
TEM: Transmission electron microscopy
UV-Vis: UV-visible
