# Supplementary material for: Repeated oral administration of low doses of silver in mice: tissue distribution and effects on central nervous system
Source: Part Fibre Toxicol. 2021 Jun 16;18:23. doi: 10.1186/s12989-021-00418-x (PMC8207582; doi:10.1186/s12989-021-00418-x)
Supplement: Supplementary file 1 — Additional file 1: Figure S1. Brain, hippocampus, immunohistochemistry for albumin. [file 12989_2021_418_MOESM1_ESM.docx]

**SUPPLEMENTARY MATERIAL**

**Additional file 1.**

**
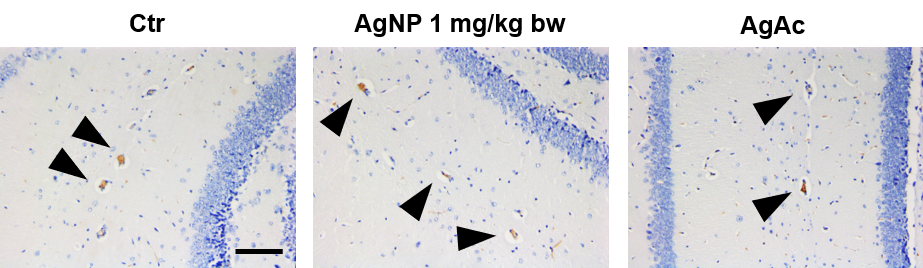
**

**Figure S1.** Brain, hippocampus, immunohistochemistry for albumin. Immunostaining of albumin in control (Ctr), AgNP 1 mg/kg bw and AgAc treated mice at the EoT, 200x (Scale bar 100 µm). Immunostained albumin within blood vessels (arrowheads). No signs of albumin extravasation were observed in treated mice.
