# Supplementary material for: Repeated oral administration of low doses of silver in mice: tissue distribution and effects on central nervous system
Source: Part Fibre Toxicol. 2021 Jun 16;18:23. doi: 10.1186/s12989-021-00418-x (PMC8207582; doi:10.1186/s12989-021-00418-x)
Supplement: Supplementary file 2 — Additional file 2: Figure S2. Representative TEM images of hippocampal synapses and myelin sheaths at the end of treatment. [file 12989_2021_418_MOESM2_ESM.doc]

**SUPPLEMENTARY MATERIAL**

**Additional file 2.**

**
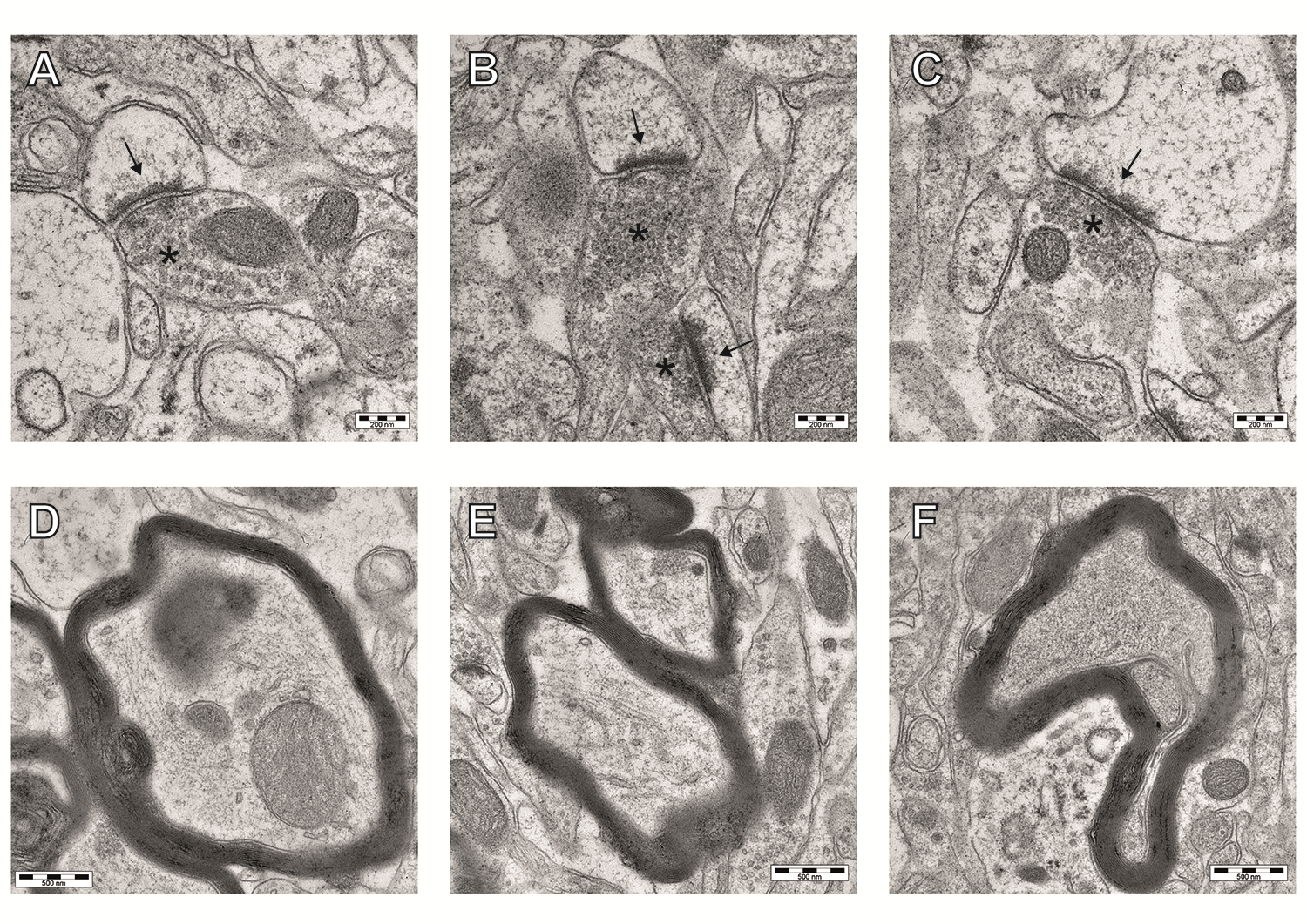
**

**Figure 2S.** **Representative TEM images of hippocampal synapses and myelin sheaths at the end of treatment.** Synapses with distinct synaptic cleft, normal appearance of postsynaptic density (black arrows) and a proper distribution of synaptic vesicles (black asterisks) were observed in control mouse (A), AgNP 1 mg/kg bw-treated mouse (B) and AgAc-treated mouse (C). Myelin sheaths wrapped around axons were formed by compacted multilamellar layers without interruptions or separations, showing comparable thickness in control mouse (D), AgNP 1 mg/kg bw-treated mouse (E) and AgAc-treated mouse (F).
